# Recruiting general practitioners for palliative care research in primary care: real-life barriers explained

**DOI:** 10.1186/s12875-019-0930-y

**Published:** 2019-03-05

**Authors:** B. Leysen, B. Van den Eynden, A. Janssens, J. Wens

**Affiliations:** 1 Department of Primary and Interdisciplinary Care, Faculty of Health and Life Sciences, University of Antwerp, Universiteitsplein 1, 2610 Wilrijk, Antwerp Belgium; 2 Multidisciplinary Pain Centre, Antwerp University Hospital, Antwerp, Belgium; 3 Department of Thoracic Oncology, Antwerp University Hospital, Antwerp, Belgium

**Keywords:** Implementation, Palliative care, Pathway, Primary care, Action research

## Abstract

**Background:**

The implementation of early palliative care within a primary care setting is a recent academic topic. Recruiting General Practitioners (GPs) to participate in a palliative care study can be challenging. The pro-Spinoza project implemented a Care Pathway for Primary Palliative Care in 5 areas in Belgium. During this project, the feasibility of the recruitment of GPs and palliative care patients was evaluated.

**Methods:**

The recruitment process was recorded in detail via an electronic logbook combining quantitative and qualitative data. Quantitative recordings included the contact types and the number of contacts with eligible GPs and were analysed descriptively. Qualitative recordings included field notes with feedback from the GPs and other stakeholders and were thematically analysed starting from the Grol and Wensing framework for professional behaviour change.

**Results:**

Of 4065 eligible GPs working in 5 areas under research, 787 GPs (19%) were contacted individually, 398 GPs (9,8%) were contacted face-to-face and most of these 398 GPs showed high interest in the topic. 112 GPs (2,8%) signed the collaboration agreement, but finally only 65 GPs (1,6%) delivered at least a completed baseline-questionnaire. Despite the initial interest in participating, the unpredictable and busy daily workloads of the GPs, as well as inexperience with research protocols, impeded the ability of the GPs to fully engage in the study. This resulted in the high dropout rate. Participating GPs reported that they had underestimated the effort required to effectively participate in the project.

**Conclusions:**

Recruitment of GPs to palliative care research is challenging. Primary care is a vital service to engage in palliative care research however the practical limitations reduce the ability of the service to effectively engage in the research. More research is needed to determine how GPs might be better supported in research.

**Trial registration:**

ClinicalTrials.gov, NCT02266069, Registered 16th October 2014, retrospectively registered.

## Background

Palliative care is considered to be a public health issue which deserves to be tackled by a systemic approach, including primary care services [1]. In Belgium, the average size of the patient population per full-time General Practitioner (GP) is approximately 1000 persons [2]. With a mortality of 1% per year [3], this equates to 10 patients per GP per year. Of those, 9 patients will experience an “expected” death [4] and would meet the criteria for palliative care services. People who are eligible for palliative care often present with complex needs [5]. However, the prevalence of palliative care needs in the general population is low [6, 7]. For this reason, many patients are not identified timely and as a result, die in hospital rather than in a location of their choice [5, 8].

GPs are often the first point of call for patients with these complex symptoms and so, high quality palliative care is only achieved if the primary care professionals can recognise this need and also work in collaboration with palliative care specialists [9].

Creating sustainable models for palliative care is a difficult task, for which in a few countries pioneers have tried to pave the path [10, 11]. One of these pioneering projects was the Pro-Spinoza project [12], sponsored by the Belgian National Institute for Health and Disability Insurance (NIHDI), which aimed to evaluate the implementation of a Care Pathway for Primary Palliative Care (CPPPC) in five areas in Belgium: two Dutch-speaking areas (1 and 4), two French-speaking areas (2 and 5) and a bilingual area 3. These five areas were delineated by the working territories of collaborating palliative care networks (PCNs). In every area of Belgium, a PCN is designed for general coordination and linkage of relevant local palliative care services. Within these networks a palliative home care team (PHCT) is designed to offer concrete help and advice to primary care professionals when they are confronted with complex palliative care situations.

In primary care research, it has previously been acknowledged that there are two main barriers to successful recruitment: prevalence of eligibility and agreement to participate. Primary care teams typically have small numbers of eligible patients for any study. And all primary care teams have to decide separately whether they want to participate in any study [13]. An additional barrier to recruitment is that GPs and other health care professionals within a primary care setting often refuse participation in research focused on palliative care [14, 15].

In palliative care research, recruitment process outcomes are seldom presented in detail, even though knowledge of this process can fundamentally influence research outputs and facilitate recruitment in future studies [16].

Therefore, this study aims to evaluate the feasibility of the recruitment of GPs and palliative care patients for the evaluation of the CPPPC.

## Methods

### Summary of the pro-Spinoza project

Participating GPs were asked to provide palliative care according to the principles of the CPPPC, in which they were trained by the research team in educational sessions [12]. The main principles were early identification of all patients older than 45 years (with cancer, dementia, organ failure, or frailty) at risk of palliative care needs using the Surprise Question (SQ) [17] and/or the Supportive and Palliative Care Indicator Tool (SPICT) [18], early assessment of needs and wishes (including advance care planning) and recognizing the early, transitional and dying stages combining the Palliative Performance Scale (PPS) [19] with clinical knowledge. This means that the proposed intervention differed significantly from usual care, in a context where even the national benchmarks for palliative care are only about terminal cancer patients [20].

Participating GPs were also asked to perform three research tasks [12]:

1. Filling a baseline-questionnaire online, including a ‘practice denominator’. Because Belgium has no patient lists within primary care, we asked participating GPs to mark all patients seen in ten consecutive days. Gender, age, location of contact (surgery, patient’s home, or residing institution) and answer to the SQ [17] (life expectancy more of less than 1 year) were registered. This task could be done prospectively or retrospectively.
2. Including at least one patient: asking a palliative care patient and his or her (in)formal caregiver to sign a participation agreement and deliver contact data (email addresses) of both patient and (in)formal caregiver. This allowed them to complete online questionnaires on quality of life and (palliative) care received.
3. Filling online questionnaires: prospectively the PPS [19] monthly for ‘focus’ patients, and retrospectively a self-evaluation of the GPs’ care for an included patient one month after this included patient had died.

The specific objectives of the whole pro-Spinoza study were [12]:

1. To reduce hospital deaths from a rate of 50% down to 35% (primary outcome);
2. To direct use of services during the last year of life towards quality-of-life (secondary outcome);
3. To record quality of life and quality of care as perceived by patients, (in)formal caregivers and GPs (descriptive);
4. To monitor the level of implementation of the CPPPC by the GPs (descriptive);
5. To understand the circumstances (how and why) in which the implementation of the CPPPC works or does not work.

Thanks to the stepped wedge cluster design used in this study, with 5 areas (=clusters), 5 steps and 1 baseline measurement, the required sample size of GPs was approximately 180, thus 36 GPs per area [12]. Two project facilitators (2*0,3 FTE) were employed for the participant recruitment phase.

### Setting and sample

The Belgian health care system is very liberal, with freedom of choice for patients and therapeutic freedom for health care professionals [21]. 3% of all Belgians choose to be attended by a GP working in a capitation payment system, while 97% of Belgians choose to visit a GP on a fee-for-service basis [2]. Except accreditation, peer-review and non-coercive feedback on therapeutic profiles, GPs don’t have incentives for quality-directed care [22].

In every part of Belgium, there are single-handed GP practices (with only one GP), mono-disciplinary GP group practices (only GPs, and sometimes a secretary) and multidisciplinary GP group practices (GPs and nurses, sometimes other professions), with varying proportions per area. Rural areas, which are most common in the French-speaking southern part of Belgium, contain more single-handed practices than group practices - an international phenomenon [23]. Urban environments contain more GP group practices and particularly more multidisciplinary practices. These varying proportions of types of practices per area suggested the need for an adapted strategy per area in recruiting GPs for this project.

The start of CPPPC implementation per area was in January 2014 for Area 1, in June 2014 for Area 2, in September 2014 for the Dutch-speaking GPs in Area 3, in April 2015 for Area 4, in June 2015 for the French-speaking GPs in Area 3 and in September 2015 for Area 5.

### Recruitment logic

Two of the most important anticipated bottle-necks in this project were to convince GPs to participate in the project and to make them encourage palliative care patients and their family members to participate in the project and deliver the necessary data.

The collaborating PCNs were asked by the researchers to work closely together with the local GPs’ circles for the local coordination of this research project. GP circles are territory-based groups of 50 to 150 GPs working together to organize out-of-hours services, continued medical education and representational activities with local partners and authorities.

After a large-scale regional educational session (3–4 h) demarcating the start of the implementation in the area, the PCN and the research tried to set up many small-scale educational sessions (1–2 h) and office visits to find GPs interested in participation.

### Activities to recruit GPs

All GPs (*n* = 4065) working in the five areas received an invitation to participate letter by post from the awarding authority, i.e. the NIHDI, who stressed the importance of the project. The PCNs helped to spread the message for recruitment into the project through the GP circles and through their direct communication with local GPs. Many GPs in the 5 project areas received several messages on the same topic through different channels.

In addition to these letters and messages, the research facilitators visited many GP practices. These visits to GPs’ practices (30–90 min) can be divided in two types: presentations at GP team meetings and visits to individual GPs. Both types of office visits were mostly preceded by both a live information session (educational session or a focus group), and at least one contact by mail and/or phone to arrange the office visit. Exceptions were a group practice and a single-handed GP both in Area 4 who requested a project presentation on receiving the NIHDI-letter.

Individual GPs were selected for office visits in three different ways:

1. During education sessions, by asking GPs to write their name on the ‘interest list’;
2. By asking chairmen of GP circles who of their GP members would be interested;
3. During routine calls by a thoracic oncologist with the GP of patients with metastatic lung cancer (only in Area 1).

Although focus groups and regional project follow-up meetings were not designed as recruitment activities, the GPs who attended these sessions were informed about the project and this sometimes motivated for participation in a visit to a GP practice to discuss possible participation of the GP in the project.

A summary of GP recruitment activities and their results can be found in Table 1 (phone calls and emails not included). As explained above the areas started implementing the project in chronological order; Area 5 had much less time than Area 1 to organize activities.

**Table 1:** Overview of GPs’ participation in recruitment activities per project area

	Area 1	Area 2	Area 3	Area 4	Area 5
Focus groups (participants)	2 (7)	1 (7)	2 (15)	1 (9)	1 (3)
Large scale educational sessions (total participants, GP participants)	3 (145, 45)	1 (50, 19)	2 (57, 42)	1 (27, 27)	Organized, but only 3 GPs interested: finally cancelled.
Small scale educational sessions (GP participants)	8 (86)	1 (21)	6 (64)	1 (7)	0
Regional meetings (GP participants)	5 (31)	1 (2)	2 (13)	2 (26)	1 (5)
Office visits at GP team meeting (GP participants)	8 (35)	1 (4)	3 (6)	4 (18)	0
Office visits to individual GPs	17	40	15	11	14

### The logbook: following-up progress of interested/participating GPs

A worksheet in MS Excel was developed to be a logbook in which all contacts with all correspondents were noted, besides all remarks. For the research team it was both an address book and a shared diary. The observations served as a basis for the ongoing reflection on the improvement of the recruitment process. This logbook offered the opportunity to register many details of all possible encounters and contacts with GPs and other stakeholders: whether they were already informed, interested, participating or collecting data.

This logbook gave insights in the recruitment and data collection process both in a quantitative way (e.g. number of phone calls, and personal contact moments needed before agreement to participate), and in a qualitative way (reasons for refusal, doubt and/or agreement to participate).

The first author included data describing the recruitment process before October 2014 of his own diary in retrospect into the logbook. From October 2014 onwards all data in the logbook were registered prospectively, mainly by the project facilitators.

### Analysis of qualitative data

Data collection occurred by two project facilitators during the project, where the analyses and discussions were performed by the whole research team.

First, the data from the Dutch speaking GPs were analysed using the principles of thematic analysis, starting from Grol and Wensing’s theory of professional behaviour change in which five steps are recognized: Orientation, Insight, Acceptance, Change, Maintenance [24]. After discussion within the team, a consensus on the codes was established. The first author used these consensus codes to analyse the feedback of the French speaking GPs as well, to be sure that no new information would be missed.

## Results

### Signature success per recruitment activity

Table 2 summarizes the success ratios of the different types of recruitment activities. Here, recruitment success is defined as GPs signing the collaboration agreement of the project.

**Table 2:** Success rate of recruitment activities for signature by GPs of collaboration agreement

	Number of attending GPs	Prevalence of attending GPs informed before event (%)	Prevalence of attending GPs who signed agreement directly after event (%)	Prevalence of attending GPs showing interest, leading to more activities (%)
Focus groups	41	8/41 (19,5%)	5/41 (12,1%)	2/41 (4,8%)
Large scale educational sessions	133	0	2/133 (1,5%)	22/133 (16,5%)
Small scale educational sessions	178	16/178 (8,9%)	2/178 (1,1%)	35/178 (19,7%)
Regional meetings	77	39/77 (50,6%)	1/77 (1,3%)	15/77 (19,5%)
Office visits at GP team meeting	59	12/59 (20,3%)	25/59 (42,4%)	0
Office visits to individual GPs	97	17/97 (17,5%)	48/97 (49,5%)	17/97 (17,5%)

The conversations held during recruitment activities often showed the high level of interest of GPs in this topic. Moreover, of 97 GPs visited individually in their office, 48 signed the agreement to participate in the project. However, as described in Table 3, this was not often followed by a commitment to the project.

**Table 3:** Overview recruitment process per project area and in total^a^

	Area 1	Area 2	Area 3	Area 4	Area 5	Total
Total number of GPs^b^	649 (100%)	500 (100%)	1561 (100%)	760 (100%)	595 (100%)	4065 (100%)
Number of GPs individually contacted^c^ (% of total number of GPs)	151 (23%)	103 (20%)	452 (29%)	52 (6,8%)	29 (4,9%)	787 (19%)
Number of GPs contacted face-to-face^d^ (% of total number of GPs)	128 (19%)	89 (18%)	130 (8%)	30 (3,9%)	21 (3,5%)	398 (9,8%)
Number of GPs having signed^e^ (% of total number of GPs)	43 (6,6%)	19 (3,8%)	22 (1,4%)	25 (3,3%)	3 (0,5%)	112 (2,8%)
Number of GPs delivering data^f^ (% of total number of GPs)	25 (3,8%)	6 (1,2%)	12 (0,7%)	21 (2,7%)	1 (0,2%)	65 (1,6%)
Number of GPs including patients (% of total number of GPs)	6 (0,92%)	2 (0,40%)	1 (0,06%)	3 (0,39%)	0	12 (0,29%)
Number of patients^g^	6	5	1	11	0	24

^a^ In the interpretation of these results it is important to remember the differences in time frame, with Area 1 having the longest time for implementation and Area 5 the shortest. See ‘Summary of the pro-Spinoza project’

^b^ All GPs received a letter from the NIHD, and many GPs received a mail from their PCN and/or GP circle

^c^ Contact via individualised email, phone call or large scale educational session

^d^ Contact via focus group, small scale educational session (< 15 participants), or office visit

^e^ GPs who signed the agreement to participate, regardless of effective participation

^f^ GPs who delivered at least one content, mostly their base line questionnaire

^g^ Of the 24 patients included, 15 were included by 3 very active GPs in Areas 2 and 4, the other GPs included only 1 patient. For 16 patients, the main pathology is known: 4 with chronic heart failure, 4 with dementia, 4 with frailty, 3 with terminal cancer and 1 with mixed cardiorespiratory failure

### Recruitment per area

Table 3 shows in a quantitative way how GPs progressed through different stages of the recruitment and data collection process..

As shown in Table 4, of the 112 GPs having signed the collaboration agreement, 85 GPs had needed one to three contacts (mail, phone call or face-to-face meeting) with a recruiter before signing. The other GPs needed more contacts before signing.

**Table 4:** Numbers of contacts needed before signature of agreement, per project area and in total

# contacts^a^	Area 1	Area 2	Area 3	Area 4	Area 5	Total
1 ^b^	12		1	8		21
2	12	9	3	1	2	27
3	15	7	6	8	1	37
4	3	3	5	3		14
5			6	3		9
6	1			1		2
7			1			1
9				1		1

^a^ # contacts: number of contacts needed before signature of agreement

^b^. GPs who signed after a single contact, signed directly after a GP team meeting, except two GPs in Area 1 who signed directly after a large educational session

### A special recruitment activity

In the University Hospital of Area 1, a well-motivated thoracic oncologist initiated a palliative care consultation within 12 weeks after a patient received diagnosis of lung cancer. After this consultation, the patient’s GP was contacted via telephone [25]. During these routine calls to GPs the pro-Spinoza project was also discussed. In doing this, 9 interested GPs were identified. Of these 9 GPs, 4 participated in the study, and 3 included a patient. This recruitment strategy was the most interesting in terms of research efficiency compared to the other recruitment approaches. We can postulate that the reason for this success was because the project information was delivered specifically at the time when a palliative care patient from the practice had been identified.

### Understanding doctor’s barriers for participation

In the qualitative research performed on the logbook, these were the main themes emerging, as categorized according to the five-step model of professional behaviour change [24]:

1. Orientation: the difficulties in contacting GPs, irrespective of the contact method (phone, mail, face-to-face contact) e.g.
  - Letters sent to local GPs by palliative care networks were noticed or read by only a few GPs;
  - The high number of phone calls necessary before a GP would be open to discussing the study;
  - GPs often mention they are overworked, particularly at the time of flu epidemics *“The flu is in the country. Can you please call me back in one month?”* (male GP, Area 2);
  - GPs often complained that participating in research will not help them for accreditation;
  - An absolute lack of interest in research: *“I never participate in research, because that is against my principles”* (male GP, Area 1)
2. Insight: the defensive attitude of GPs to change their palliative care practice, e.g. these reasons:
  - *“I do not have patients in palliative care right now”* (female GP, Area 1);
  - *“How can I have anticipatory care planning talks with all my pre-palliative care patients?”* (female GP, Area 1);
3. Acceptance: whilst initially interested in the study, many GPs struggled with accepting the practical and logistical implications involved with participating. GPs identified the following barriers why they could not start participating:
  - Illness of a colleague working in the same practice;
  - Competition with other running studies in the primary care setting, with GPs admitting they don’t want to participate in too many studies at the same time or shortly after each other *“The flue is here and I have already participated in other studies”* (male GP, Area 4);
  - Communication skills: a difficulty many GPs face in delivering bad news, especially in a ‘grey zone’ of advanced chronic disease leading to an early palliative care phase.
4. Change: the GPs having fully accepted to try out the change by participating in the project still had to run through a phase of fitting this goal into reality. In this phase, the obstacles proved to be from a practical nature and in interaction with the context (patients and their family members, paramedics), mostly in real confrontation with time management and man power, e.g.
  - Complexity of the project: Three GPs committed formally before they pulled back from participation, because the practical barriers proved to be more difficult to handle than initially thought;
  - Time constraints: although filling out the email derived questionnaire just took a few minutes, some participating GPs admitted they never succeeded in doing so. “*I can find myself in your project, but I don’t find the time for it. My colleagues in the practice are still participating and I am supporting them wherever I can. Can you please delete me from your list?*” (female GP, Area 4);
  - Online data collection difficult for patients: including suitable patients for the project seemed to be challenging in practical terms as well. A participating GP, who tried to convince other GPs to participate in the project complained about the new methodology used within the project: “*In theory I know some patients who can be included, but in practice this proves to be difficult, because neither they nor their family can use internet easily and the nursing home personnel is not really interested to put effort in data collection for this project. That is why I am still looking for a suitable candidate.”* (male GP, Area 4)*;*
  - Online data collection difficult for GPs: many GPs stated that to upload research data online was a barrier for them.

The fifth step in the Grol and Wensing model is ‘Maintenance’, but that stage was not the focus of this project and was not discussed during the recruitment phase.

Except the barriers clearly related to the five-step-model, other themes also emerged:

1. Procrastination in all phases e.g.:
  - On signing the collaboration agreement: *“You will laugh with me, but I still have not had a look at it”* (male GP, Area 4). The project facilitator suggested that the GP might want to put a self-imposed deadline for the task. *“In one week, also on Thursday evening”.* The next phone call with this GP was for the definitive answer for this phase*: “I cannot participate in the study there is too much work in the practice and at the moment my wife is ill. But congratulations with the study, it is a very good initiative that I can appreciate”*.
  - On filling the base line questionnaire: *“You are right, the paper work still has to be done, but next month I will do what I can*.” (female GP, Area 1)
2. Disorganisation hindering all phases, e.g.:
  - A GP asked the project facilitator to call her back one week later and to send her an email with more details. She had lost her papers (given to her one week earlier). The facilitator emailed her about the project and never got a response after this.
  - Many times, GPs seemed to have disappeared, because their phones rung without being picked and their emails remained unanswered. This also happened with already participating GPs.
  - Many times GPs denied to have received a certain email which was certainly sent by the research team, not more than one week earlier.
3. The belief that research is an additional and unwanted burden for palliative care patients and their caregivers: *“I don’t see the added value of participation for my palliative care patients.”* (male GP, Area 1).

There was no substantial difference between the barriers described by Dutch speaking GP and by French speaking GPs.

## Discussion

### Main findings

The aim was to evaluate the feasibility of recruitment of GPs and palliative care patients for the CPPPC project (pro-Spinoza). The main barriers for GPs were linked to the wide range of activities that were asked from them when participating in the pro-Spinoza study: clinical actions, administrative actions and having to discuss the research with patients to ask them to participate. If the last two tasks can be taken over maximally by researchers, this could help to identify more GPs and patients willing to participate..

Recruiting GPs for palliative care research, and through them their patients, requires a lot of time and effort, and can best be planned beforehand with local palliative care leaders and general practice leaders. In order to participate most GPs have to hear about it at least two or three times, preferably in their own office in a one-to-one conversation. A time efficient recruitment strategy includes:

1. Large and small scale educational sessions to inform and train GPs;
2. Lists of interested GPs made by
  - asking GPs directly at these educational sessions;
  - asking local GP and palliative care leaders which GPs could be interested;
  - detecting patients who may benefit from (early) palliative care and asking their GPs whether they are interested;
3. Finally visits to GPs’ offices to motivate them individually for the project.

The concrete barriers for participation described in this study led the research team to state that implementation strategies should be directing at all five steps of the Grol and Wensing model plus general time management skills. With general time management skills is meant ‘being reachable’ and ‘do what you say, say what you do’. One of the keys to successful recruitment is to make sure that GPs see the extra value of participating in the project, either for themselves (such as accreditation) or for their patients (improved end of life care).

### Adapting the recruitment strategy during the project

The recruitment strategy as presented in this article was not clear from the beginning of the project. Through constant monitoring and ongoing reflection based on the information in the logbook, innovations in the recruitment strategy occurred. The stepped wedge design, starting the project in another area every 6 months, allowed the research team to learn from previous experiences before starting up in the next area.

Some lessons learnt and concrete solutions in this project are summarized here:

- A large-scale educational session can work for dissemination of a concept, but not for implementation of a project. That is why the research started to perform visits to GPs’ offices, to motivate them after they had been informed;
- Performing visits to each GPs’ office is time consuming. That is why the research facilitators were hired in the middle of the project;
- Many GPs were convinced of the importance of a project only after having heard of it in different ways, particularly when the message comes from respectable sources. That is why NIHDI-letters started being sent to the GPs in the middle of the project;
- For many GPs, the difference between the proposed CPPPC and routine palliative care was too large to implement the CPPPC fully. That is why during the project, the research team decided to focus on early identification of (cancer or non-cancer) palliative care patients, on breaking bad news in a sensitive way, and on advance care planning - with the message that particularly in early palliative care advance care planning is possible even without talking about death;
- GP groups who collectively signed often failed to be compliant to the project, so that strategy was abandoned in the end, in favour of recruiting individual GPs;
- The opportunity to collaborate with the thoracic oncologist came in the middle of the project and was gladly accepted by the research team;
- In the last months of the study GPs were more strictly selected on their level of motivation (as perceived by the research facilitators) before the research team invested in activities to recruit them.
- During the study, research facilitators took over more and more data collection tasks from GPs, wherever possible.

Before the pro-Spinoza project, the CPPPC had been piloted in a small-scale setting with some Belgian Dutch-speaking practices [26] and was approved by most participating GPs to be feasible. However, because the researcher could follow the participants more closely in the pilot project than in the pro-Spinoza study, these GPs probably had more support in using the CPPPC and less administrative research burden than in the pro-Spinoza study.

It is clear that the pro-Spinoza was overambitious and would probably have benefited from a more limited clinical scope of early identification of palliative care needs and starting advance care planning. What would also have helped is a more limited research burden for the GPs.

### Compared with literature

A Dutch primary palliative care randomized clinical trial shows, similarly to the results in our study, a high interest of GPs in early palliative care research and a high drop-out rate of trained GPs. Only 28 of 57 trained GPs included patients, and only 77 of 134 participating GPs (intervention and control group together) delivered data [27].

In a recent Italian study (Arianna) [28], 94 GPs identified 937 patients with a low life expectancy and potential needs compatible with palliative care, and they were followed-up prospectively. Some strengths present in this Italian study could have made the difference:

- Integration of data collecting software into the standard clinical folders of the GPs;
- Including a multidimensional evaluation through validated tools by the home palliative care unit (HPCU) of patients reported by GPs, with the goal of integration of primary care and HPCU care.

In further communication with the study authors, other aspects were identified. In a context where one of the main Italian scientific societies for GPs promoted courses in palliative care and developed a profile of ‘GPs with a Special Interest in palliative care’, they applied these three recruitment strategies:

1. Selecting 10 GPs who were trained to be ‘GPs with Special Interest in palliative care’ and who were supported and motivated during the entire project. These 10 GPs worked in 10 different areas of Italy. During the project, each of these 10 GPs became a point of reference and support for 9 other GPs in their area.
2. Basing the selection of these 100 GPs on strict criteria, all compulsory but one:
  Having participated in the preceding Italian implementation project for GPs on palliative care and pain management (Teseo)(single non-compulsory criterion);
  Express the commitment to collaborate with the associated HPCU of the area;
  Using the specific medical software adopted in the Arianna project to share data;
  Participating in the training course and in periodic meetings;
  Serving at least 1000 patients.
3. Granting the GPs a certain amount of resources for the extra activities performed, at the end of the project when they had proved their fulfillment of the protocol.

First detecting patients potentially benefiting of an intervention and then recruiting their GPs is proven to be effective in end-of-life research. In an Australian study focusing on general practice needs-based care for carers of people with advanced cancer, 155 of the 158 GPs whose patient had agreed to participate decided to participate themselves [29].

Hospital-based recruitment is often more successful than recruitment in primary care settings. Only 4% of the referrals to a palliative care intervention in a Norwegian randomized clinical trial occurred by the community clinics, and the great majority of referrals occurred by hospital doctors [30].

In our study, one of the main obstacles reported in the logbook was the GPs’ belief that research is an additional and unwanted burden on patients and their family members. This belief should lead to a careful design of any palliative care research project and sharing with GPs that there is evidence that patients and their family members like to participate in research, rather than discouraging palliative care patients to participate in research [31].

General suggestions for successful patient recruitment in end-of-life care research are among others: a dedicated study nurse [30, 32, 33], systematic screening of patient lists [34], having the support of clinical champions [30, 33, 34], personal contacts between recruiters and participating doctors rather than giving information on paper [30], using simple referral routines [30], flexibility in recruitment to accommodate patients’ needs [30, 34], taking enough time for the recruitment period [33], training of GPs in the attitude and skill necessary to incorporate research in sensitive care processes [33], ‘careful messaging’ in person rather than by telephone [30], basing key messages of the recruitment visit on known barriers for palliative care research [33], thoughtful messaging to make research relevant [34], highlighting positive aspects of the participant-researcher relationship [31] and to help others in the future in similar situations [31], and if possible having additional resources of a trials cooperative group [34].

In our study, a main barrier both for recruitment of GPs and for inclusion of palliative care patients by GPs was the anticipated fear of having an anticipatory care planning conversation – even though all GPs received training on this aspect before signing the collaboration agreement. One answer to this fear could be the concept of integrated palliative care, in which professional networking is deemed more important than standardisation of care [35]. A qualitative study found that stakeholders were much more concerned with how primary care providers would navigate the post-identification period than with early identification itself.

Implications of early identification include the need for a team- based approach to identification and to engage broader communities to ensure people live and die well post-identification [36]. This means that a supportive professional network in which the GP feels safe to work with palliative care patients can enhance self-efficacy in identifying and following up palliative care patients and that this network is more important for a good outcome than having a guideline available.

### Strengths and limitations

This study highlights that complementary quantitative and qualitative methods can achieve a very detailed description of recruitment efforts for a palliative care intervention in the primary care setting– which is a difficult research area in terms of recruitment and attrition. This has been done for five areas, which makes the study a multiple case study [37]. Although the five areas differ in language and health care culture, the basic principles of recruitment for primary palliative care research seem to apply to all five areas. The stepped wedge design allowed for learning from experiences in initial areas, who had implemented the project earlier, to inform the recruitment in the following areas.

Limitations are that, although the data was collected prospectively, an underreporting bias is likely to have happened since the greatest focus of the research team was to recruit GPs (and subsequently patients) more than reporting the most rigidly possible on the recruitment process. In other words, the actual study was a “by-product” of the main study project, although it proved to be one of the most insightful parts of this main study. Other limitations were the lack of support that could be given to the participating GPs and the many differences between usual palliative care and the proposed CPPPC. A project with a smaller scope could have been more successful. The analysis of the qualitative data happened by one researcher with consensus forming within the team afterwards for the data of Dutch-speaking GPs, and subsequently by only one researcher for the data of French-speaking GPs.

### Implications for policy and research on primary palliative care

In Belgium, orientation [24] of GPs towards implementation of innovations could be fostered by including more incentives for quality-directed care and research activities in the already existing accreditation process and by including (palliative) care benchmarks in the feedback GPs receive from the NIHDI on their therapeutic profile.

Primary care research in general can be boosted by using routinely collected sociodemographic and clinical data, to help universities and government bodies to understand how the field is handling changing care needs, and to help them to give feedback to the field [38].

Primary palliative care research in particular will benefit from fitting research projects into daily practice: detecting patients who need (early) palliative care, and telling the GP how to help these patients achieve the best possible treatment with the help of the researchers and local palliative care leaders. What would also be beneficial is creating trials cooperative groups focusing on primary palliative care.

Based on this study, it is recommended that, when setting up more implementation research projects of early palliative care in primary care settings, it is necessary to do so with special attention for the recruitment process. It can be interesting to investigate which local system-based approaches can be beneficial for the general promotion of early palliative care. For instance, based on our limited experience with hospital-based identification of palliative care patients, a transmural collaboration for early palliative care could be set up. Nursing homes and home care nurses could also select patients at-risk to help GPs in detecting eligible patients for early palliative care. Besides, further research is necessary on the most effective and efficacious recruitment methods for GPs and patients in primary care. This study taught us that recruitment strategies themselves are worth of further carefully designed investigation. Besides patient identification and recruitment strategies, also the feasibility of different data collection methods might be an interesting topic for further research in optimizing participation in primary palliative care research.

## Conclusions

Recruitment in primary palliative care research is difficult for many reasons. This study showed a high level of interest from the GPs to be involved in the study, but also produced high GP dropout rates and low data completion. Suggestions for future recruitment and quality improvement efforts in this field are formulated. The key is to offer a well-defined intervention which clearly benefits the GPs and/or the patients and a well-designed research protocol which takes the research burden as much as possible away from GPs and patients.

## Abbreviations

CPPPC: Care Pathway for Primary Palliative Care
GP: General practitioner
HPCU: Home palliative care unit (Italy)
NIHDI: National Institute for Health and Disability Insurance (Belgium)
PCN: Palliative care network (Belgium)
PHCT: Palliative home care team (Belgium)
PPS: Palliative Performance Scale
SPICT: Supportive and Palliative Care Indicator Tool
SQ: Surprise Question

## References

[1] World Health Organisation (2014). Strengthening of palliative care as a component of comprehensive care throughout the life course. 67th world health assembly.

[2] InterMutualistic Agency. The InterMutualistic Agency Atlas. Available at: http://atlas.ima-aim.be/databanken. Accessed 3 Mar 2019.

[3] Death statistics in Belgium, available at https://statbel.fgov.be/nl/themas/bevolking/sterfte-en-levensverwachting/sterfte#figures/. Accessed 3 Mar 2019.

[4] Lunney J, Lynn J, Hogan C (2002). Profiles of older Medicare decedents. J Am Geriatr Soc.

[5] Beynon T, Gomes B, Murtagh F, Glucksman E, Parfitt A, Burman R, et al. How common are palliative care needs among older people who die in the emergency department? 10.1136/bmjspcare.2009.090019rep 24653232

[6] Gómez-Batiste X, Martínez-Muñoz M, Blay C, et al. Identifying patients with chronic conditions in need of palliative care in the general population: development of the NECPAL tool and preliminary prevalence rates in Catalonia. BMJ Support Palliat Care. 10.1136/bmjspcare-2012-000211. 10.1136/bmjspcare-2012-000211 24644748

[7] Mason B, Boyd K, Steyn J, Kendall M, Macpherson S, Murray SA. Computer screening for palliative care needs in primary care: a mixed-methods study. Br J Gen Pract 2018;68(670):e360-e369. 10.3399/bjgp18X695729. Epub 2018 Mar 26. 10.3399/bjgp18X695729 PMC5916083 29581129

[8] Agentschap Zorg en Gezondheid (Agency Care and Health). Place of death in Flanders, Belgium (2016). https://www.zorg-en-gezondheid.be/plaats-van-overlijden-2016. Accessed 3 Mar 2019.

[9] Quill A (2013). Generalist plus specialist palliative care – creating a more sustainable model. N Engl J Med.

[10] Petrova M, Dale J, Munday D, Koistinen J, Agarwal S, Lall R (2010). The role and impact of facilitators in primary care: findings from the implementation of the gold standards framework for palliative care. Fam Pract.

[11] Gómez-Batiste X, Murray SA, Thomas K, Blay C, Boyd K, Moine S, et al. Comprehensive and integrated palliative Care for People with Advanced Chronic Conditions: an update from several European initiatives and recommendations for policy. J Pain Symptom Manag 2017 Mar;53(3):509–517. doi: 10.1016/j.jpainsymman.2016.10.361. Epub 2016 Dec 30. 10.1016/j.jpainsymman.2016.10.361 28042069

[12] Leysen B, Van den Eynden B, Gielen B, Bastiaens H, Wens J. Implementation of a care pathway for primary palliative care in 5 research clusters in Belgium: quasi-experimental study protocol and innovations in data collection (pro-SPINOZA). BMC Palliative Care. 2015;14:46. 10.1186/s12904-015-0043-x. 10.1186/s12904-015-0043-x PMC4585994 26416574

[13] Hanratty B, Lowson E, Holmes L, Addington-Hall J, Arthur A, Grande G (2012). A comparison of strategies to recruit older patients and carers to end-of-life research in primary care. BMC Health Serv Res.

[14] Bower P, Wallace P, Ward E, Graffy J, Miller J, Delaney B, Kinmonth AL. Improving recruitment to health research in primary care. Family Practice Advance Access. 2009. 10.1093/fampra/cmp037. 10.1093/fampra/cmp037 19549623

[15] Ewing G, Rogers M, Barclay S, McCabe J, Martin A, Todd C. Recruiting patients into a primary care based study of palliative care: why is it so difficult? Pall Med. 2004;18:452–9. 10.1191/0269216304pm905oa 15332423

[16] Rhondali W, Berthiller J, Hui D, Yennu S, Lafumas V, Ledoux M (2013). Barriers to research in palliative care in France. BMJ Support Palliat Care.

[17] Pattison M, Romer AL (2004). Improving care through the end of life: launching a primary care clinic-based program. Journal of Palliative Medicine July.

[18] Highet G, Crawford D, Murray S, Boyd K. Development and evaluation of the supportive and palliative care Indicator tool (SPICT): a mixed-method study. BMJ Support Palliat Care. 2013. 10.1136/bmjspcare-2013-00048. 10.1136/bmjspcare-2013-000488 24644193

[19] Victoria Hospice (Canada). Palliative Performance Scale (PPS). Available at https://www.victoriahospice.org/sites/default/files/ppsv2_english_-_sample_-_dec_17.pdf. Accessed 3 Mar 2019.

[20] Belgian Health Care Knowledge Centre. Performance of the Belgian health system – report 2015. (KCE Report 259C) 2016. Available at https://kce.fgov.be/sites/default/files/atoms/files/KCE_259C_performancereport2015_0_0.pdf. Accessed 3 Mar 2019.

[21] Belgian Ministry of Health. Overview of the Belgian health care system. https://www.health.belgium.be/en/health/taking-care-yourself/patient-related-themes/cross-border-health-care/healthcare-providers. Accessed 3 Mar 2019.

[22] European Observatory on Health Systems and Policies. Assuring the quality of health care in the European Union – a case for action. Observatory Studies Series N° 12. 2008. ISBN 978 92 890 7193 2. http://www.euro.who.int/__data/assets/pdf_file/0007/98233/E91397.pdf?ua=1. Accessed 3 Mar 2019.

[23] Boerma WGW. Profiles of general practice in Europe. NIVEL. 2003; https://nvl004.nivel.nl/nivel-2015/sites/default/files/bestanden/profiles-of-general-practice-in-europe.pdf. Accessed 3 Mar 2019.

[24] Grol R, Wensing M. What drives change? Barriers to and incentives for achieving evidence-based practice. Med J Aust. 2004;180(6 Suppl):S57. https://www.mja.com.au/system/files/issues/180_06_150304/gro10753_fm.pdf. Accessed 3 Mar 2019. 10.5694/j.1326-5377.2004.tb05948.x 15012583

[25] Janssens A, Teugels L, Kohl S, Michielsen T, Leysen B, Van Meerbeeck J (2016). Practical tools for implementing early palliative care in advanced lung cancer. Eur Respir J.

[26] Waerenburgh C, Streffer ML, Antonneau F, Van den Eynden B, Wens J, Remmen R (2010). Ontwikkeling van een Zorgpad voor het Naderende Levenseinde in de eerste lijn. Voorbereidende studie [Development of a Care Pathway for the Nearing End-of-life in primary care] Antwerp.

[27] Thoonsen B, Vissers K, Verhagen S (2015). Training general practitioners in early identification and anticipatory palliative care planning: a randomized controlled trial. BMC Fam Pract.

[28] Scaccabarozi G, Amodio E, Pellegrini G, Limonta F, Lora Aprile P, Lovaglio PG (2018). The “ ARIANNA” project: an observational study on a model of early identification of patients with palliative care needs through the integration between primary care and Italian home palliative care units. J Palliat Med.

[29] Mitchel GK, Girgis A, Jiwa M, Sibbritt D, Burridge LH, Senior HE (2013). Providing general practice needs-based care for carers of people with advanced cancer: a randomised controlled trial. Br J Gen Practice.

[30] Jordhøy MS, Kaasa S, Fayers P, Underland G, Ahlner-Elmqvist M. Challenges in palliative care research; recruitment, attrition and compliance: experience from a randomized controlled trial. Palliat Med. 1999;13(Issue 4):299–310. 10.1191/026921699668963873. 10.1191/026921699668963873 10659099

[31] Samar A, Slayer S, Deas K, Nekolaichuk C (2016). Family caregiver participation in palliative care research: challenging the myth. J Pain Symptom Manag.

[32] Leblanc T, Lodato J, Currow D, Abernethy A (2013). Overcoming recruitment challenges in palliative care clinical trials. J Oncol Practice.

[33] Ruijs C, Goedhart J, Kerkhof A, Van der Wal G, Onwuteaka-Philipsen B (2011). Recruiting end-of-life cancer patients in the Netherlands for a study on suffering and euthanasia requests. Fam Pract.

[34] Hanson L, Bull J, Wessell K, Massie L, Bennett R, Kutner J (2014). Strategies to support recruitment of patients with life-limiting illness for research: the palliative care research cooperative group. J Pain Symptom Manag.

[35] Den Herder-van der Eerden M, van Wijngaarden J, Payne S, Preston N, Linge-Dahl L, Radbruch L, et al. Integrated palliative care is about professional networking rather than standardisation of care: a qualitative study with healthcare professionals in 19 integrated palliative care initiatives in five European countries. Palliat Med. 32(6):1091–102. 10.1177/0269216318758194. 10.1177/0269216318758194 PMC5967037 29436279

[36] Urquhart R, Kotecha J, Kendell C, Martin M, Han H, Lawson B, et al. Stakeholders’ views on identifying patients in primary care at risk of dying: a qualitative descriptive study using focus groups and interviews. Br J Gen Pract. 2018:bjgp18X698345. 10.3399/bjgp18X698345. 10.3399/bjgp18X698345 PMC6104853 30104331

[37] Yin R. Case study research: design and methods. SAGE publications; 2009. ISBN 9781412960991.

[38] Delaney B, Peterson K, Speedie S, Taweel A, Arvantis T, Hobbs R (2012). Envisioning a learning health care system: the electronic primary care research network, a case study. Ann Fam Med.

